# Does sample rate introduce an artifact in spectral analysis of continuous processes?

**DOI:** 10.3389/fphys.2012.00495

**Published:** 2013-01-21

**Authors:** Maarten L. Wijnants, R. F. A. Cox, F. Hasselman, A. M. T. Bosman, Guy Van Orden

**Affiliations:** ^1^Behavioural Science Institute, Radboud University NijmegenNijmegen, Netherlands; ^2^Department of Developmental Psychology, Heymans Institute, University of GroningenNetherlands; ^3^Cap Center for Cognition and Action, University of CincinnatiCincinnati, OH, USA

**Keywords:** 1/*f* noise, 1/*f* scaling, spectral analysis, periodic sampling, sample rate

## Abstract

Spectral analysis is a widely used method to estimate 1/*f*^α^ noise in behavioral and physiological data series. The aim of this paper is to achieve a more solid appreciation for the effects of periodic sampling on the outcomes of spectral analysis. It is shown that spectral analysis is biased by the choice of sample rate because denser sampling comes with lower amplitude fluctuations at the highest frequencies. Here we introduce an analytical strategy that compensates for this effect by focusing on a fixed amount, rather than a fixed percentage of the lowest frequencies in a power spectrum. Using this strategy, estimates of the degree of 1/*f*^α^ noise become robust against sample rate conversion and more sensitive overall. Altogether, the present contribution may shed new light on known discrepancies in the psychological literature on 1/*f*^α^ noise, and may provide a means to achieve a more solid framework for 1/*f*^α^ noise in continuous processes.

Over recent decades, there has been an increasing interest in the time-evolutionary properties of psychological data series, and the number of methods to quantify the *degree-of-randomness* in time series data is rapidly expanding. It is becoming increasingly acknowledged that the variation from one measurement to the next rarely fluctuates randomly, as traditionally assumed in most standard statistical methods (Gilden et al., 1995; Gilden, 2001; Van Orden et al., 2003). Especially the presence of 1/*f* noise (also called 1/*f* scaling or pink noise) in repeated performances is a robust finding. The presence of 1/*f* noise implies that a data signal may not be accurately described without incorporating time at the level of analysis. We will first explain the workings of spectral analysis through a fictive example, and then we explain how spectral analysis can be used to estimate the presence of 1/*f* noise.

Consider a participant, performing a 500-trial simple response task. The task instruction is, for instance, to press a button whenever a stimulus is presented. The dependent variable of interest for the researcher is response time to the stimulus. This participant's average response time turns out to be 500 ms with a standard deviation of 35 ms. However, this participant's task performance constitutes the unrealistic case where the pattern of response variability over time looks exactly like a sine wave (see Figure 1A). Now, imagine another participant, who received the same task instruction, and showed exactly the same response times but in a different trial order (see Figure 1C). While both response series have an identical mean and standard deviation, they show a distinct pattern of responses over time.

**Figure 1:**
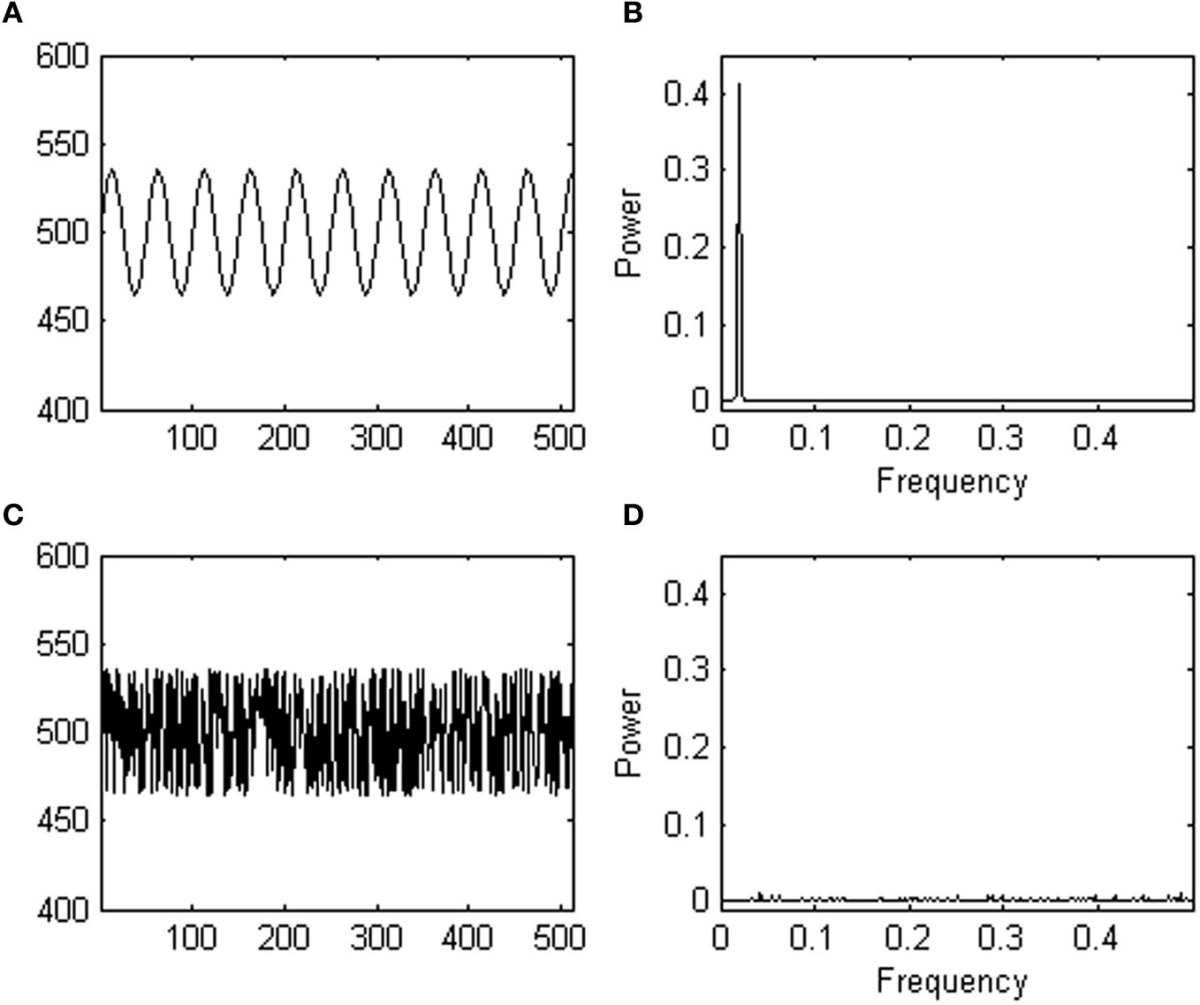
**(A)** Shows a fictive data series yielding response times oscillating as a sine wave (in milliseconds, y-axis) over trials (x-axis). **(B)** Shows a power spectrum of the fictive data series shown in **(A)**; note the peak. **(C)** Shows the same data series as in **(A)** after randomization. **(D)** Shows a power spectrum of the randomized data series shown in **(C)**; note the absence of a peak.

Statistics based on central tendency measures are not sensitive to the different pattern of variability observed in both participants. If in one experimental group all participants were like participant 1, and in another experimental group all participants were like participant 2, a *t*-test for instance, would not differentiate among both groups because the groups would yield equal means and standard deviations. Yet, a different inherent process likely produced the responses. Thus, a researcher may wonder whether trial-to-trial fluctuations observed in an experiment occur randomly or not, and ask whether there is anything systematic about the observed temporal patterns of variation.

Spectral analysis is one of the available methods to estimate the degree of randomness in a pattern of responses over trials. Spectral analysis translates dependencies in the time domain (i.e., a pattern of change in response time over trials) as simple features in the frequency domain using an operation called a Fourier transform, which decomposes the data series containing changes in response over trials into its constituent frequencies. Next, the power (the square of the amplitude) at each frequency in the decomposed signal is plotted in a so-called a power spectrum (also called power spectral density function). For instance, a power spectrum of participant 1′ s response series (shown in Figure 1B) reveals one peak at the dominant frequency of the sine wave. Participant 2′ s responses do not yield a dominant frequency in the time domain, and consequently a spectral analysis does not reveal any peaks in the power spectrum (see Figure 1D). Thus, while the performances of both participants are indistinguishable using central tendency measures, the two different temporal arrangements of the same responses are distinct in the frequency domain. The power spectrum thus provides information which effectively complements information from *t*-tests, ANOVA's, etc. (see Slifkin and Newell, 1998; Riley and Turvey, 2002, for more examples).

Spectral analysis can not only be used to detect simple periodicities as in the example above, but can also be used to quantify more complex and realistic patterns of variation in psychological data series. Consider, for instance, another participant in the simple reaction task whose response times show a pattern of variability called 1/*f* noise, as shown in Figure 2A. 1/*f* noise is a complex sequence effect spanning over the entire time course of an experiment, and comprises undulating “waves” of relatively longer and then shorter response times that travel across the series. In a 1/*f* signal, faster (high-frequent) changes in response time are typically small, and embedded in overarching, slower (lower-frequent) changes of higher amplitude. In only a few simple steps, this characteristic pattern of response variability can be observed through spectral analysis. First, a Fourier transform translates the data series into the sum of sines and cosines that best fits the data series. This is schematically represented in Figure 2B. Next, the frequency and power (amplitude^2^) of each of the fitted waveforms are plotted against each other in a power spectrum (see Figure 2C). Figure 2D shows the power spectrum on log-scales, which makes the 1/*f* noise pattern even more visible; power is in inverse proportion to frequency. The log–log power spectrum in Figure 2D yields a slope of −1 (hence, 1/*f*^1^ noise).

**Figure 2:**
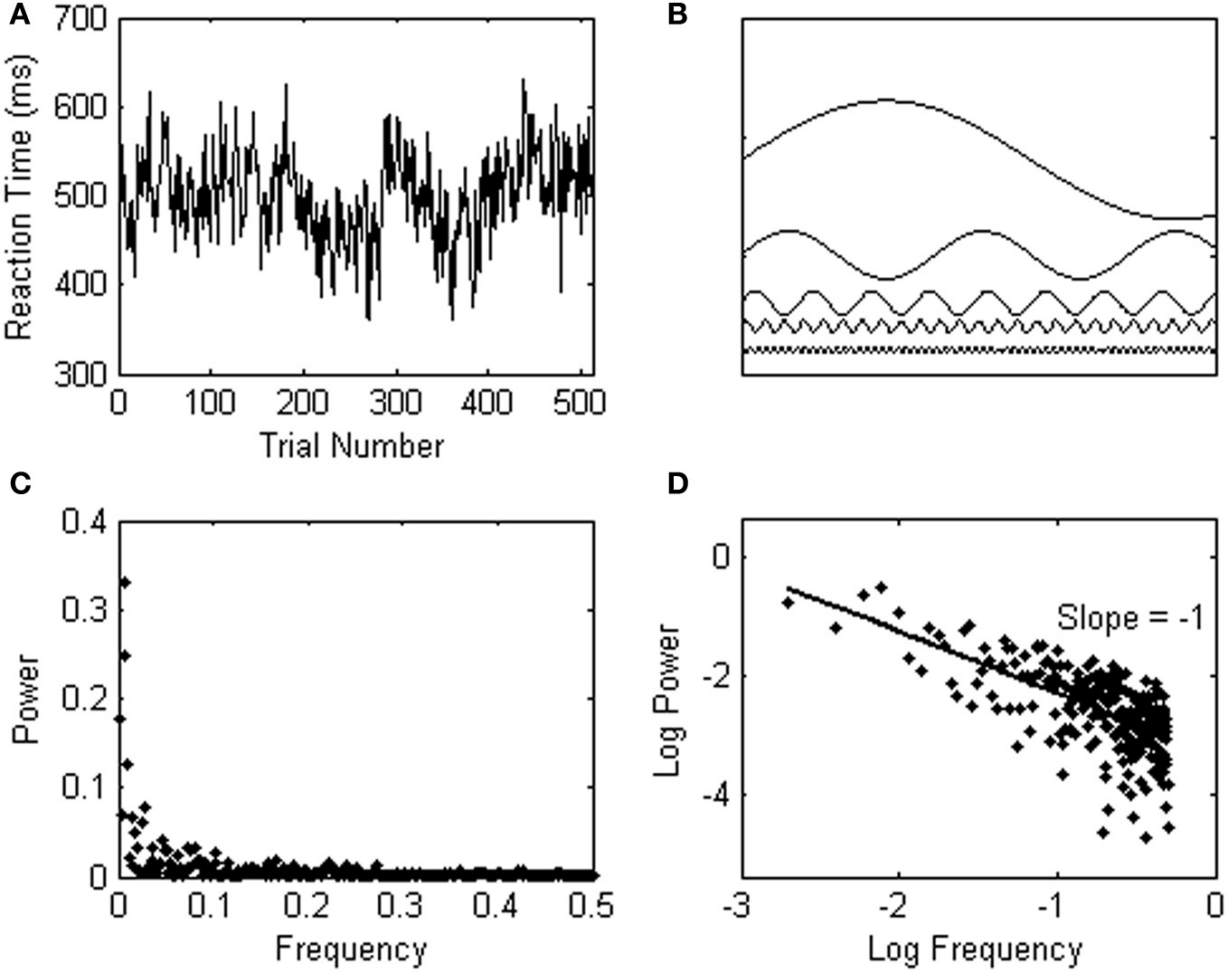
**(A)** Shows a response series yielding 1/*f* noise. **(B)** Schematically represents a number of sine waves which are fitted to the data series through a Fourier transform. **(C)** Shows the 1/*f* noise pattern in a power spectrum, which is shown on logarithmic scales in **(D)**.

Observing 1/*f* noise may run against standard statistical intuitions because the variability in psychological data is usually assumed to fluctuate randomly from trial to trial. A data series with random background noise (also called white noise, see Figure 3A), however, does not yield a relationship among frequency (*f*) and a particular change of amplitude S(*f*) in the signal (see Figure 3B). A power spectrum of white noise variability has a flat slope on log scales (yielding 1/*f*^0^ noise).

**Figure 3:**
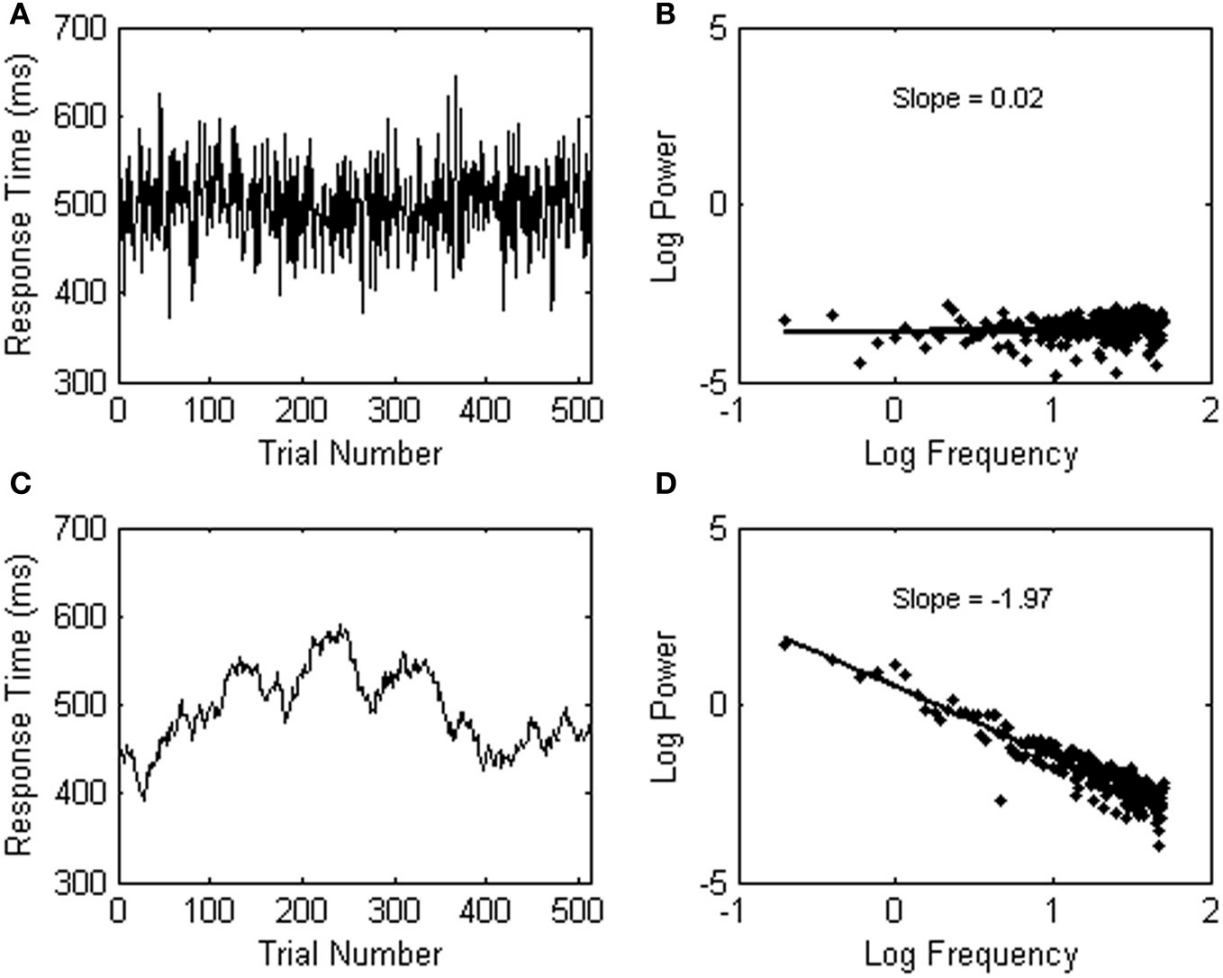
**(A)** Shows an example of white (random) noise. The power spectrum of the white noise series is shown in **(B)**. **(C)** Shows an example of Brownian noise. The power spectrum of the Brownian noise series is shown in **(D)**.

A third category of noise is called Brownian noise (see Figure 3C), and can be described as 1/*f*^2^ noise (see Figure 3D; the slope is −2). Brownian noise is also called a random walk, because it can be produced by adding a random increment to each sample to obtain the next. In contrast to white noise, which can be produced by randomly choosing each sample independently, Brownian noise yields persistence or memory in the data series.

## 1/f noise in human performance

1/*f* noise has been observed in repeated responses in many cognitive tasks. Examples include simple and choice reaction (Kello et al., 2007), mental rotation (Gilden, 1997), visual search (Aks et al., 2002), lexical decision (Gilden, 1997), word naming (Van Orden et al., 2003), color and shape discrimination (Gilden, 2001), implicit associations (Correll, 2008), and self-reports of self-esteem (Delignières et al., 2004), to name a few examples. Apart from the ubiquitous presence of 1/*f*-like noise in cognitive performances (Kello et al., 2007), 1/*f* noise has been observed in temporal patterns of variation at all levels of neural (Werner, 2010) and physiological organization (West, 2010).

The origins of 1/*f* noise in human cognition remain a theoretical topic of debate, however, (Van Orden et al., 2003, 2005; Wagenmakers et al., 2005; Torre and Wagenmakers, 2009; Diniz et al., 2010). Nonetheless, the relative presence of 1/*f* noise (hence, the slope −α) has empirically been shown to distinguish among experimental conditions (Kello et al., 2007; Diniz et al., 2010; Van Orden et al., 2011, are reviews). Therefore, the slope of a power spectrum is an informative measure in psychological research. The scaling exponent α in 1/*f*^α^ noise usually varies between white noise and 1/*f* noise (0 < α < 1), but sometimes also between 1/*f* noise and Brownian noise (1 < α < 2).

Intriguingly, empirical evidence has accumulated suggesting that the relative presence of 1/*f* noise is related to the coordination of cognitive and physiological processes. For instance, deviations from 1*/f* noise (either toward white noise or toward Brownian noise) have been found with epilepsy (Ramon et al., 2008), heart failure (Goldberger et al., 2002), fetal distress syndrome (Goldberger, 1996), major-depressive disorder (Linkenkaer-Hansen et al., 2005), mania (Bahrami et al., 2005), attention-deficit-hyperactivity-disorder (Gilden and Hancock, 2007), developmental dyslexia (Wijnants et al., 2012b), autism (Lai et al., 2010), Alzheimer's disease (Abásolo et al., 2006), Huntington's disease (West, 2006), and Parkinson's disease (Hausdorff, 2007). In addition, the presence of 1/*f* noise correlates, for instance, with the severity of depression symptoms (Linkenkaer-Hansen et al., 2005), the success rate of recovery from traumatic brain injury (Burr et al., 2008), and falling risk in elderly (Hausdorff, 2007). Also, the presence of 1/*f* noise increases with learning (Wijnants et al., 2009) and may decrease as task demands increase (Clayton and Frey, 1997; Correll, 2008). In each case the overly random or overly rigid behaviors showed a value of α further from 1, compared to conditions allowing for more flexibly stable and adaptive performances.

These studies confirm the importance of time series methods like spectral analysis in psychological research. Interestingly, however, all of the examples above are based on the analysis of *trial series* or interval series. In a trial series, each sampled data value represents a measure of a discrete response or response interval, as in the example of the simple reaction task mentioned earlier. Many variables in psychological research, however, are continuous in nature, rather than discrete. Continuous processes are represented as a *time series* through periodic sampling. Periodic sampling means that the continuous process *x* → (*t*) is digitized as a sequence of discrete data values *t*_1_, *t*_2_, *t*_3_, *t*_n_ …, where the total number of data points depends on the chosen sampling rate. Interestingly, however, the clear framework suggested by the role of 1/*f*^α^ noise in trial series has not (yet) found a univocal parallel in the analysis of psychological time series.

Here, we investigate whether differences in sample rate constitute an artifact which obscures comparisons across studies and experimental conditions. The paper is organized as follows. First, a number of details pertaining to analytical choices for spectral analysis are discussed. Then, it is discussed in which way sample rate affects the frequency content of a time series, and it is explained how this artifact is usually dealt with in psychological studies of 1/*f*^α^ noise relying on continuous processes. Next, we show how this approach renders heterogeneous estimates of the slope −α, and offer an alternative solution that circumvents the artifact.

## 1/*f* noise and periodic sampling

Psychologists are in general well-aware of the characteristics of a desired sampling regime. That is, any signal that has been periodically sampled can only be perfectly reconstructed if the sampling rate corresponds to a frequency that is minimally twice the highest frequency in the original signal (this is known as the Shannon–Nyquist sampling theorem; Shannon, 1949). When sampling more sparsely, a phenomenon called aliasing is likely to occur. Aliasing means that fluctuations outside of the measured frequency range are misinterpreted as different frequencies that fall within the measured range of frequencies, yielding distorted results (see Holden, 2005). Therefore, sample rate is an important input parameter when applying spectral analysis to periodically sampled data series. The estimated frequencies should not be faster than half the sample rate. For example, when a given time series is sampled at 100 Hz, the frequencies estimated in spectral analysis (the x-axis in the power spectrum) should fall in the range of 0–50 Hz to avoid aliasing.

The next input parameter for spectral analysis is the number of frequencies to be estimated within the non-aliased frequency range. This parameter will determine the number of data points in the power spectrum. A spectral analysis with maximum frequency resolution will estimate half as many frequencies as there are data points, because the highest resolvable frequency oscillates back and forth every other data point. In order to understand why the regression fit over the 25% lowest frequencies covers such a substantial portion of the power spectrum (as can be seen in Figures 2D and 3C,D), note that a Fourier transform evaluates the power of each frequency within the signal equidistantly within the desired frequency range. After the log transformation, however, the frequencies are no longer equidistant, and exponentially more frequencies are observed in the high-frequency range than in the low-frequency range of the power spectrum.

When the goal of the spectral analysis is to estimate the α scaling exponent (thus, the negative slope of the logarithmic power spectrum, or the presence of 1/*f* noise), another choice concerns the number of frequencies in the power spectrum over which the slope is fitted. That is, the slope −α is rarely fitted over all frequencies, because it is known that a power spectrum often gives unreliable results in the highest frequency range. Specifically, the right-hand side of a power spectrum often presents a flattening (or whitening) of the slope (Holden, 2005; Holden et al., 2011). Therefore, excluding the highest frequencies in the log–log regression is generally recommended (Beran, 1994; Eke et al., 2000, 2002; Holden, 2005). The linear fit is often limited to the 25% lowest frequencies that compose the spectral slope (Eke et al., 2000, 2002) or even 10% (Taqqu et al., 1995), to achieve more reliable scaling estimates of the scaling exponent α.

## The artifact of sample rate

The aim of this study is to achieve a more solid appreciation for the effects of periodic sampling on the outcomes of spectral analysis. Specifically, a researcher's choice of sample rate is known to change the estimated α exponents in a particular way (Carlini et al., 2002; Eke et al., 2002), and this bias is usually not anticipated. This is especially problematic when different studies are compared, which employ a different sampling regime of similar performances (i.e., comparing the outcomes of spectral analysis of trial series with outcomes of spectral analysis of time series), or which rely on periodic sampling but employ different sample rates.

Carlini et al. (2002) point out that higher sample rates yield steeper spectral slopes, hence larger α scaling exponents, compared with more sparsely sampled processes. “The amplitude of the (*highest frequency*) oscillations themselves decreases sharply (*when sample rate increases*)” (Carlini et al., 2002, p. 246, emphasis added for terminological consistency). Eke et al. (2002) add: “Increasing *f*_*s*_ [*sample rate*], … cannot continue beyond some upper limit for exceeding it would increase the chance that high-frequency estimates in the power spectrum would not reflect physiology (*or more generally, the process of interest*)” (Eke et al., 2002, p. 27, emphasis added).

**Figure 4:**
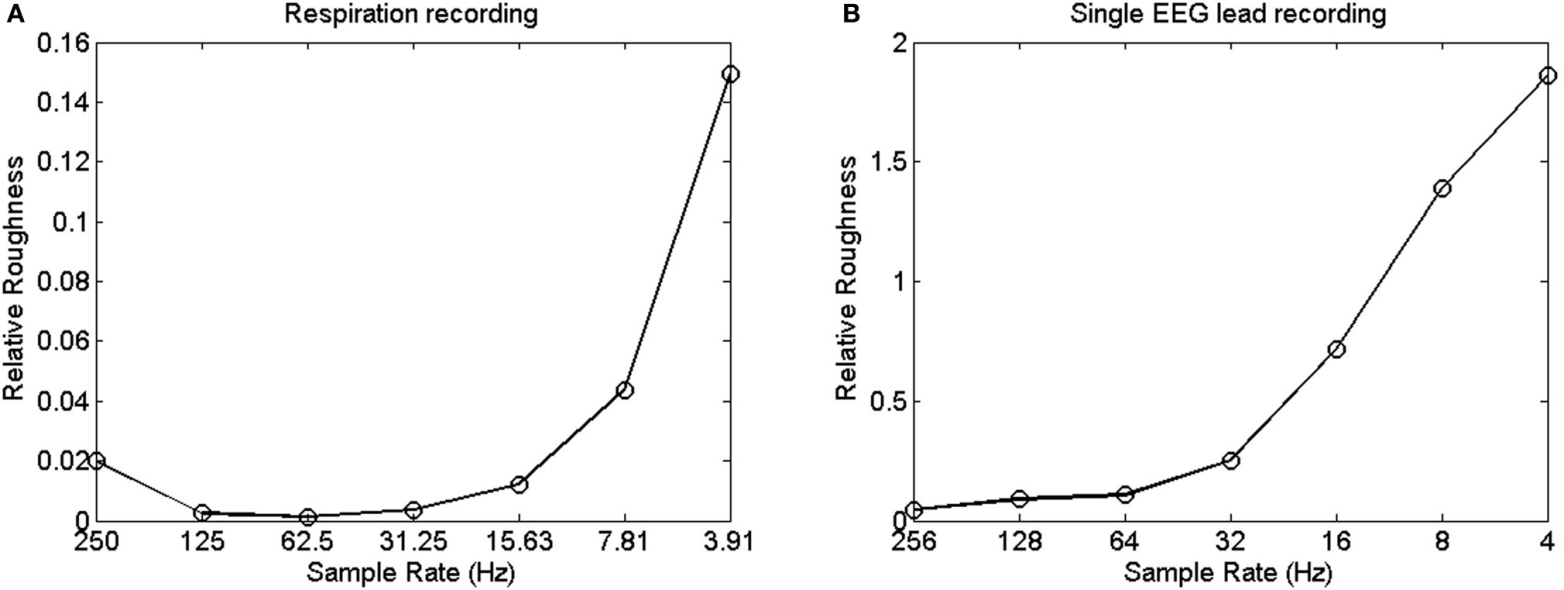
**(A)** Shows the relative roughness of a respiration time series at various sampling rates. **(B)** Shows the change in relative roughness of an EEG time series at various sampling rates.

These observations constitute the core measurement problem raised in this paper: the outcomes of spectral analysis hinge on sample rate. This artifact is visually presented in Figures 4A and B, which shows the relative roughness of two different time series (Goldberger et al., 2000) that were downsampled so that they yield different sampling rates. Relative roughness can be conceived as an index of the suitability of the monofractal framework (cf. Marmelat et al., 2012), and describes the relative contribution of local variance to the global variance of a time series. Figures 4A and B reveal that the relative roughness of a time series is reduced when sampled more densely. Specifically, Figures 4A and B suggest that faster sampling comes with lower amplitude at the higher frequencies (making the series more smooth, thus reducing local variance), which may result in overall steeper slopes in the power spectrum compared with processes that are sampled more sparsely.

This line of reasoning so far is straightforward, but can make a world of difference nonetheless concerning the utility of spectral analysis when confronted with periodically sampled, continuous processes. That is, the highest-frequency range in the spectrum has lower amplitude when higher sample rates are employed, and this artifact likely protrudes gradually into lower frequencies as sample rate further increases. Correctly, some authors have assumed that such an artifact does not affect the estimate of α, given that the biased frequencies are not used to fit the slope −α: “This would not be much of a problem if the upper 75% of the spectral estimates were to be discarded as recommended and if these irrelevant estimates would fall into the discarded range” (Eke et al., 2002, pp. 27–28). In other words, the challenge is to focus on the range of frequencies that is not contaminated by the artifact. If, however, the biased frequencies exceed the highest 75% frequency range, the assumption cited above would not be valid, and different values of α would be obtained with different sample rates. Thus, the question is whether the non-contaminated frequency range converges on the 25% lowest-frequency range.

**Figure 5:**
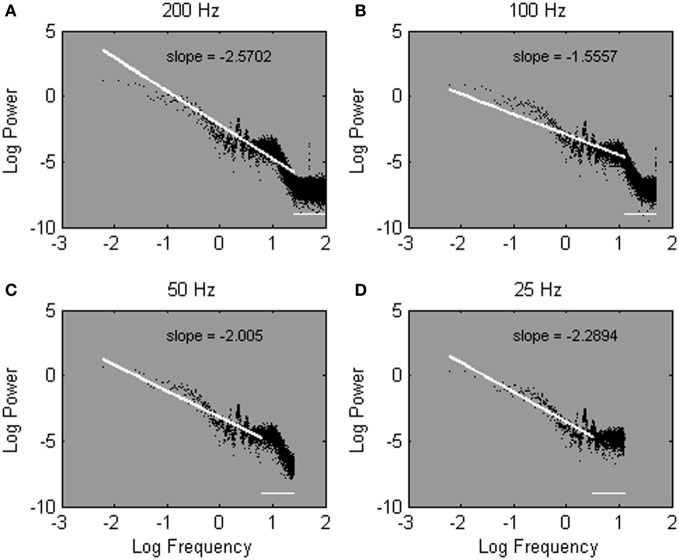
**Power spectra estimated from one Galvanic Skin Response time series sampled at 200 Hz (A), 100 Hz (B), 50 Hz (C), and 25 Hz (D).** Spectral slopes are fitted over the lowest 25% of 2^15^ **(A)**, 2^14^ **(B)**, 2^13^ **(C)**, and 2^12^ **(D)** estimated frequencies. Note that most of the estimated frequencies fall in the high-frequency range of the spectrum.

To answer the question, we evaluated a Galvanic Skin Response (GSR) time series that was sampled at either 200 Hz (yielding a time series of 2^16^ data points), 100 Hz (2^15^ data points), 50 Hz (2^14^ data points), or 25 Hz (2^13^ data points). For each sample rate of the same time series, the frequencies in the power spectrum range between 0 Hz and half the sample rate to avoid aliasing. Then, following Eke et al. (2002), the linear regression fit was plotted over the 25% lowest frequency range, to estimate α (see Figures 5A–D; the discarded 75% frequency range is represented as a horizontal line). Remarkably, Figures 5A,B show rather variable estimates of the spectral slope −α for the same measured process; α ranged between 1.56 and 2.57 depending on sample rate. In other words, even with all precautions in place, sample rate still distorts the estimate of α.

**Figure 6:**
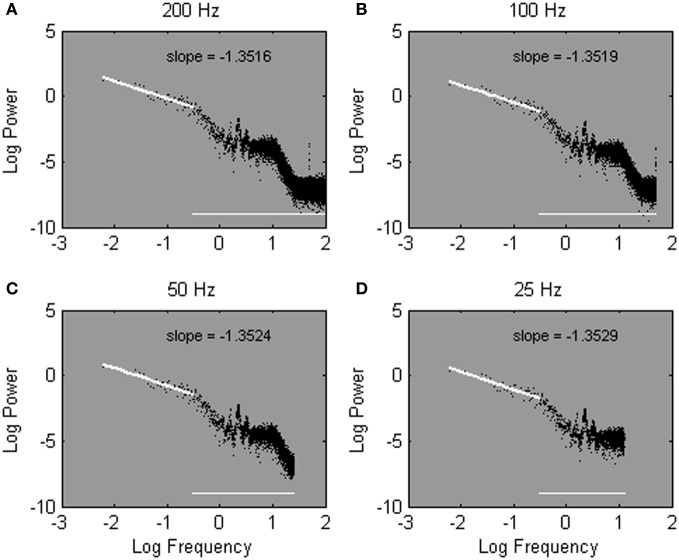
**The same power spectra as shown in Figure 5, estimated from one Galvanic Skin Response time series sampled at 200 Hz (A), 100 Hz (B), 50 Hz (C), and 25 Hz (D).** Spectral slopes are fitted over the lowest 50 of 2^15^ **(A)**, 2^14^ **(B)**, 2^13^ **(C)**, and 2^12^ **(D)** estimated frequencies.

Here, we introduce an alternative solution to the problem that outcomes of spectral analysis can hinge on sample rate. The logic is to fit the slope −α over a fixed amount, rather than over a fixed percentage, of lowest frequencies. This solution takes advantage of, rather than being contaminated by, inherent differences in sample rate. Since more frequencies are estimated overall from more densely sampled time series, fitting the slope −α over a fixed number of low-frequencies implies a fit over a lower percentage of low frequencies when a time series is sampled more densely. Thus, while the bias leaks into more of the lower frequencies for higher sample rates, a lower percentage of low-frequencies is used to fit the slope −α. At sparser sample rates, the bias extends over a smaller portion of the low frequencies, and a larger portion of estimated frequencies is used to fit the slope −α. The advantages of the introduced strategy can be seen in Figures 6A–D, which shows the same power spectra as shown in Figures 5A–D, but with the spectral slope −α now fitted over a set number of frequencies. In contrast to Figures 5A–D, robust estimates of α are obtained regardless of sample rate.

Fitting over a fixed number of frequencies is notably different from fitting over a fixed percentage of frequencies. With regard to the high-frequency range, when the slope −α is fitted over the 25% of lowest frequencies, the high-frequency range of a power spectrum is treated equally regardless of the relative presence of spurious high-frequencies, and thus, regardless of sample rate. Specifically, the range of discarded high frequencies remains equals across different sample rates. When the slope −α is fitted over a fixed number of low frequencies, as proposed here, the discarded frequency range changes as a function of sample rate. Specifically, as sample rate increases the range of discarded high-frequencies increases as well (hence, the horizontal line in Figures 6A–D). As a result, the range of discarded frequencies converges much more closely with the range of spurious frequencies.

With regard to the low-frequency range, fitting over the 25% of lowest frequencies implies fitting over a different low-frequency range for different sample rates. Specifically, relatively higher frequencies (hence, more biased frequencies) are incorporated in the fit as sample rate increases. For instance, in Figures 5A–D, the fitted frequencies range between 0 and 25 Hz, 0 and 12.5 Hz, 0 and 6.25 Hz, and 0 and 3.13 Hz for sample rates of 200, 100, 50, and 25 Hz, respectively. Fitting over a fixed amount of low frequencies (50 frequencies in this example), in contrast, implies a fit over a stable low-frequency range, regardless of sample rate. Hence, in Figures 6A–D, the cut-off frequency is the same; the slope −α is fitted between 0 and 0.31 Hz regardless of sample rate.

## Downsampling

This paper examines the artifact in the estimation of 1/*f* noise parameters introduced by the choice of sample rate. We expect, based on previous observations (e.g., Carlini et al., 2002; Eke et al., 2002), that low-amplitude fluctuations are introduced in the high-frequency range of the power spectrum as sample rate increases. We examine this artifact by comparing α exponent over a range of different sample rates using a variety of simulated and empirical time series. That is, we compare empirical or simulated data signals with their downsampled copies. In essence, downsampling is simply a *post-hoc* reduction in sampling rate by an integer factor. For a time series *x*(*n*), when downsampling by the constant factor *M*, the downsampled copy *y*(*m*) may be represented as *y*(*m*) = *x*(*nM*), where *y*(*m*) is the downsampled sequence, obtained by taking every *M*th sample from the original data sequence *x*(*n*), thereby discarding *M* − 1 samples for every *M* samples. It is to be expected that this *post-hoc* reduction in sample rate will effectively alter the spectral estimates for sampled data signals.

If increasing the sample rate has indeed the effect of reducing the amplitude of the signal at the highest frequencies, the overall estimated α exponent should increase as sample rate increases. This bias should not affect the low-frequency range of the power spectrum, and should become more pronounced when the spectral slope −α is fitted over a wider frequency range. This is investigated by fitting the spectral slope over 10, 25, or 100% of the lowest frequencies in the power spectrum. The outcomes are expected to be biased more strongly when the slope is fitted over 100% of the spectrum, and gradually become less biased as the slope is fitted over 25% (cf. Eke et al., 2002) and 10% (cf. Taqqu et al., 1995) of the lowest frequencies only. In contrast, when the slope is fitted over the lowest 50 frequencies only, and is thus fitted over a stable low-frequency range, with a stable cut-off frequency, it would be natural to expect the bias to be absent.

## The reliability of α

The empirical data series have been collected in a precision aiming study. In the study, 15 participants were invited to draw lines back and forth between two visual targets with a stylus, as fast and as accurately as possible. Participants received no instruction concerning pen pressure or pen tilt strategies. The targets were presented on a printed sheet of paper, one at the left side of the paper and one at the right side. The target width was 0.4 cm and the distance between targets was 24 cm. One block of 1100 trials was completed with the dominant hand. When the last trial was reached, a tone signaled the end of the experiment.

Pen pressure (in grams) and pen tilt (absolute deviation from the center of the stylus, in cm) coordinates were recorded using a digitizer tablet connected to a regular PC. The tablet samples at a temporal rate of 171 Hz. In addition, a GSR signal was recorded on the fingertips of the non-moving hand at 200 Hz. Also, artificial 15 white noise signals (1/*f*^0^), 15 1/*f* noise signals (1/*f*^1^), and 15 Brownian noise signals (1/*f*^2^) were generated with a series length of 2^16^ data points, using an Inverse Fourier transform algorithm described by Lennon (2000).

After data collection, each time series was prepared to fit the needs for the spectral analysis (cf. Holden, 2005). First, outliers outside 3 × the standard deviation from the mean were removed. Next, because the Fourier transform fits stationary sines and cosines to the data series, simple drifts or long-term trends may distort the results. Linear and quadratic detrending ensures that the analyzed data series is in line with the idealized mathematics of spectral analysis. Thus, linear and quadratic trends were removed for all data series (cf. Holden, 2005). Then, the original time series were normalized, and truncated by removing the data points at the beginning of the data series until 2^16^ data points were left. None of the empirical data series contained fewer than 2^16^ data values.

Next, the original data series (2^16^ data points) were downsampled by removing every next data point from the analysis, so that the new data series length was 2^15^. This procedure was iterated until only 2^10^ data points were left, thereby reducing sample rate by a factor of 2^6^. Then, for each of the resulting series, the spectral slope was either fitted over 10, 25, or 100% of the lowest frequencies, or over the 50 lowest frequencies.

### Results and discussion

**Figure 7:**
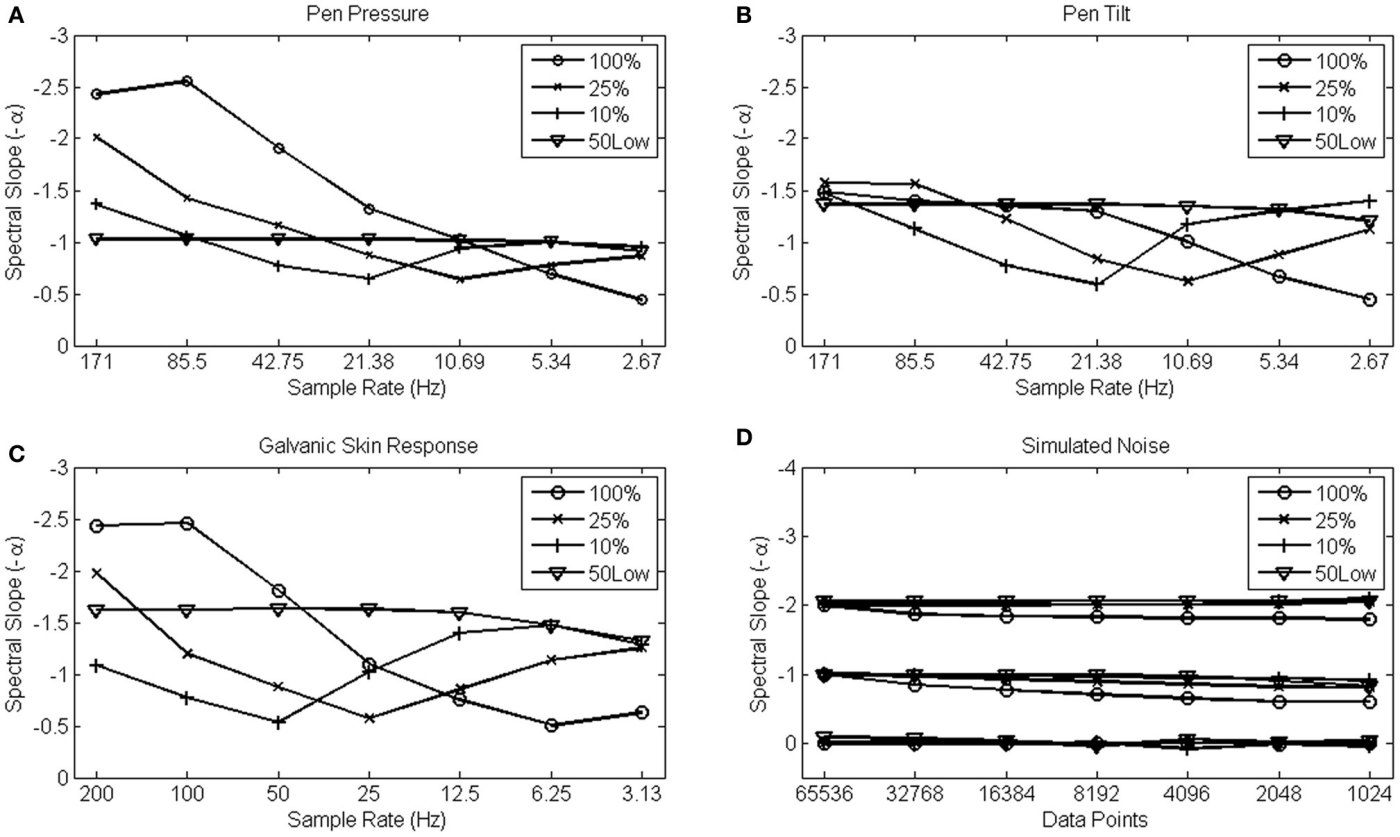
**Average α scaling exponents from 15 pen pressure (A), pen tilt (B), Galvanic Skin Response (C), and simultated 1/*f*^0^, 1/*f*^1^, and 1/*f*^2^ data series (D) are shown on the y-axis.** The x-axis shows sample rate for the empirical data series, and series length for the simulated series that also were downsampled by a factor of 2 in each step on the x-axis.

The results from the pen pressure, pen tilt, and GSR data are shown in Figures 7A–C, which represents the fitted slope −α over a range of different sample rates for each data set. The different choices of fit are shown as separate lines in each Figure. It can be seen that regardless of the percentage of low frequencies used to fit the slope −α (10, 25, or 100%), the observed α values effectively change in function of sample rate. As predicted, α exponents are higher at high sample rates. The artifact is most apparent when fitting the slope over the entire power spectrum and gradually becomes somewhat less dramatic as smaller portions of the low-frequencies are used to fit the spectral slope −α. When fitting over the 50 lowest frequencies, however (shown as 50Low in Figures 7A–D), the slope −α remains robust against sample rate conversion.

Only the pen tilt data do not entirely confirm the expected artifact. At the highest sample rates, α values derived from a fit over the entire spectrum appear more robust than α values derived from a fit over the 10 or 25% lowest frequencies. But also in this example, α values derived from a fit over the 50 lowest frequencies constituted the most robust solution.

The simulated noise patterns, however, reveal a very distinct (hence, absent) effect of sample rate. The four choices of fit that were evaluated are shown in Figure 7D for each category of noise simultaneously. The random (α = 0), 1/*f* (α = 1) and Brownian (α = 2) noise simulations reveal robust values of α, regardless the choice of fit. This result confirms that the change in α arises from differences in sample density rather than from the differences in series length *per se* (with the 100% fit somewhat less reliable than the other choices of fit, however).

These results demonstrate that the relatively arbitrary choice of a sample rate dramatically alters the value of the α exponent if the spectral slope −α is fitted over a fixed percentage of low-frequencies. The bias is so strong that sample rate appears to be more influential on the estimated exponents than the process under scrutiny itself. This artifact is obviously problematic and leaves researchers with difficult decisions concerning the reliability of their analysis. The strategy of spectral analysis introduced here results in scaling exponents that are robust against artifacts that come with dense sampling, and thus may solve those questions.

## The sensitivity of α

A final confirmation of the introduced strategy for spectral analysis would require an evaluation of the sensitivity of the estimated exponents, in addition to their robustness against sample rate conversion. Sensitive exponents are more likely to differentiate among experimental conditions, and more clearly reveal the relation among different variables, given that such relations are present. In this case, we evaluate the correlation among different streams of 1/*f* noise (pen pressure and pen tilt) that were collected simultaneously in the previously introduced motor task.

**Figure 8:**
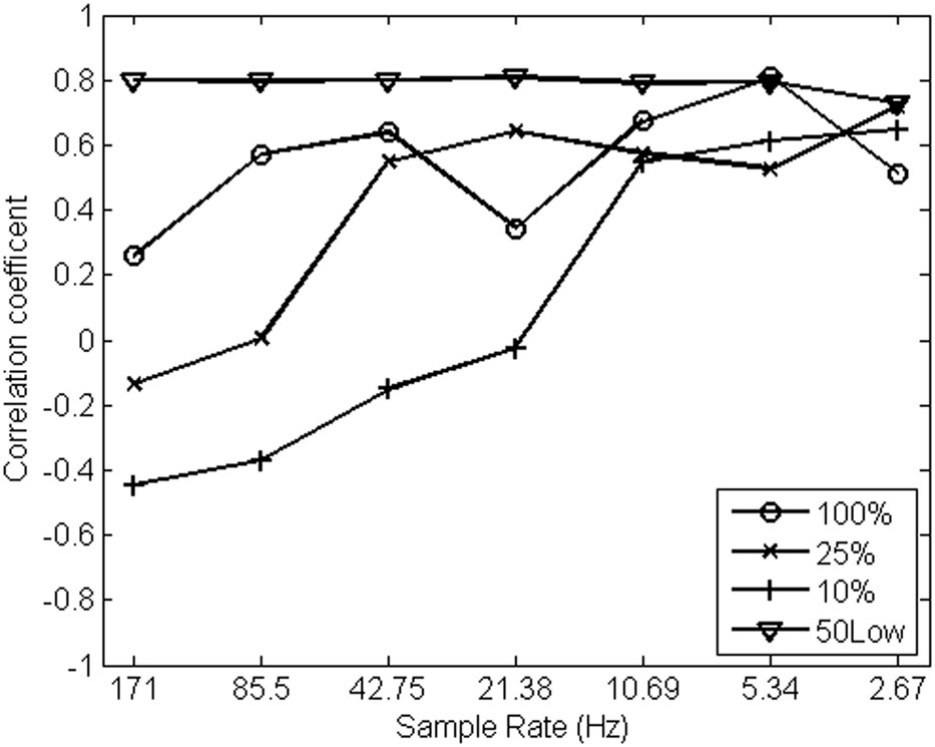
**Correlation coefficients among α exponents estimated from pen pressure and pen tilt data (y-axis, *N* = 15) over a range of sample rates (in Hz; x-axis) using different strategies for spectral analysis**.

The pattern of correlations between both streams of 1/*f* noise (pen pressure and pen tilt) shown in Figure 8 is remarkably heterogeneous over different sample rates, except for the strategy introduced here. α exponents estimated from the original, non-down-sampled data appear uncorrelated when relying on conventional spectral strategies. The correlations among pen pressure and pen tilt scaling exponents tend to grow stronger as sample rate decreases (hence, when fewer “smoothed” high-frequencies are introduced in the analysis). The introduced method for spectral analysis (shown as 50Low in Figure 8), in contrast, indicates strongly correlated streams of 1/*f* noise, regardless of sample rate.

## The validity of α

The results presented above suggest that if one follows the exact same procedure in an experimental set-up, but uses a different measurement device or device setting, one may end up with vastly deviant outcomes if sampling artifacts are not anticipated. Anticipating sampling artifacts can be as simple as fitting the power spectrum over a set number of low frequencies (i.e., fitting over a stable low-frequency range), rather than fitting the regression line over a set percentage of frequency (i.e., fitting over a variable low frequency range). This practice results in more reliable and more sensitive scaling exponents. Nonetheless, the goal should not be to fit over a prescribed amount of low frequencies (e.g., 50) *per se*. Importantly, as long as the slope does not change in function of sample rate (i.e., after downsampling), any set number will do reliability-wise. For instance; an idealized 1/*f* process would reveal a linear slope regardless of the fitted frequency range (hence Figure 7D).

Empirical data often show scale-invariance in a restricted range only, however. In these cases an optimal number of frequencies can be determined by performing a simple downsampling test (i.e., Figure 7). When the scaling outcomes do not change in function of sample rate the chosen frequency range to fit is reliable. If the outcomes do change, the number of low frequencies in the fit should be reduced until the outcomes remain robust against sample rate conversion. In this process, one should obviously be aware of two final criteria: (1) the amount of frequencies should be sufficient to yield reliable regression outcomes, and (2) a linear range of the power spectrum is preferred given the nature of the regression analysis.

With these less idealized examples of 1/*f* scaling, changing the frequency range used for slope fitting may reveal ever changing slopes over different frequency ranges, however. This would mean one would want to ascertain the validity of an estimate in addition to its reliability over different sample rates, leading to the question whether the scaling exponents derived using the suggested fitting approach are representative for the process under scrutiny.

To inquire the validity of the suggested fitting approach, we simulated artificial series using the fBmW model (Thornton and Gilden, 2005). This procedure produces series that compose a scaling part α (i.e., a fractional Brownian motion with a known exponent α) with white noise β (whose variance is β^2^) added to it. Given that relative roughness decreases at higher sampling rates (cf. Figure 4), it is fair to assume that the high-frequency range of the spectrum is an artifact of sampling, and that the valid information is to be found in the low-frequency range i.e., the alpha put in the model. In addition, faster sampling arguably is more susceptible to instrument noise that may distort spectral outcomes at the higher frequencies. Thus, power spectra produced by the fBmW-model present examples in analogy with the sampling rate artifact, producing a well-defined elbow in the power spectrum.

**Figure 9:**
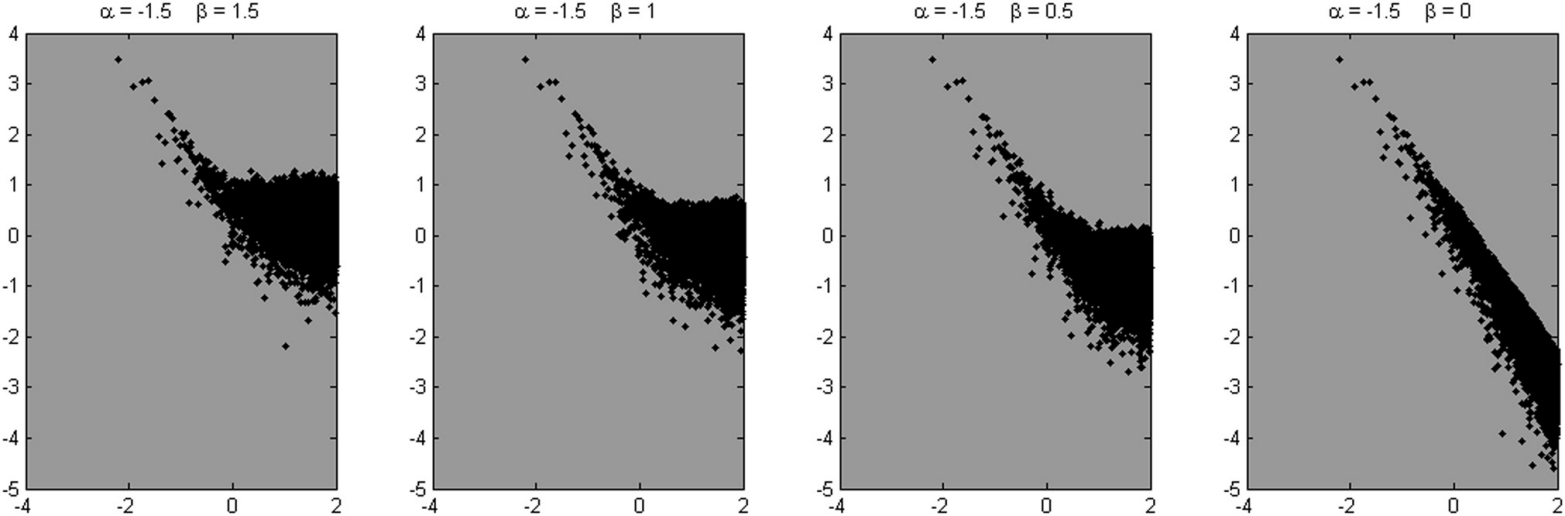
**Four example spectra are shown, derived from the signals simulated using the fBmW-model**.

**Figure 10:**
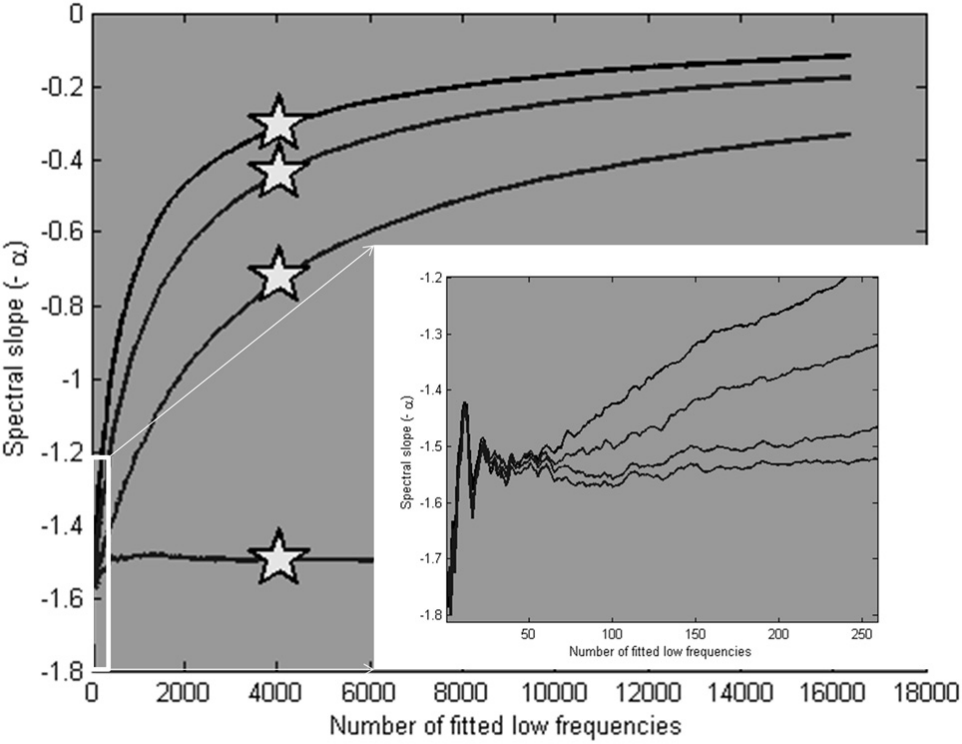
**Average scaling estimates of ten simulated signals (length is 2^16^ data points), with α is 1.5, and β is 1.5, 1, 0.5, and 0, respectively, fitted over a varying number of low frequencies.** The pentagram markers indicate the scaling estimates for a fit over the 25% lowest frequencies. In the inset, it is shown that the estimates converge closely on the modeled α parameter at around 50 low frequencies.

Four example power spectra produced by the model, with α = 1.5 and β = 1.5, 1, 0.5, and 0, respectively, are shown in Figure 9. We know from these parameters that a valid scaling estimate should approximate 1.5; a reference point against which different fitting strategies can be assessed. The x-axis in Figure 10 shows the number of low frequencies included in the regression fit. The pentagram-shaped markers indicate the exponents estimated when the spectra where fitted over 25% of lowest frequencies. The inset reveals a region of convergence around roughly 50 frequencies, after which a point of expansion reveals the white noise process added to varying degrees. This observation supports the suggestion that a fit over the lowest 50 frequencies provides valid estimates of the “true” scaling exponent α (i.e., 1.5), and questions the validity of estimates over the 25% of lowest frequencies.

This simple simulation confirms the validity of the proposed fitting strategy, but a better analogy to the empirical data is possible, however. The produced series keep a number of variables stable that vary in the empirical series (e.g., series length, number of frequencies in the spectrum, relative roughness). Also, the resulting spectra are simple in the sense that they reveal a single elbow, rather than the more complex staircase-like shape of the empirical spectra seen in Figures 5 and 6. We therefore determined empirically the parameters that resemble the empirical power spectra more closely.

**Figure 11:**
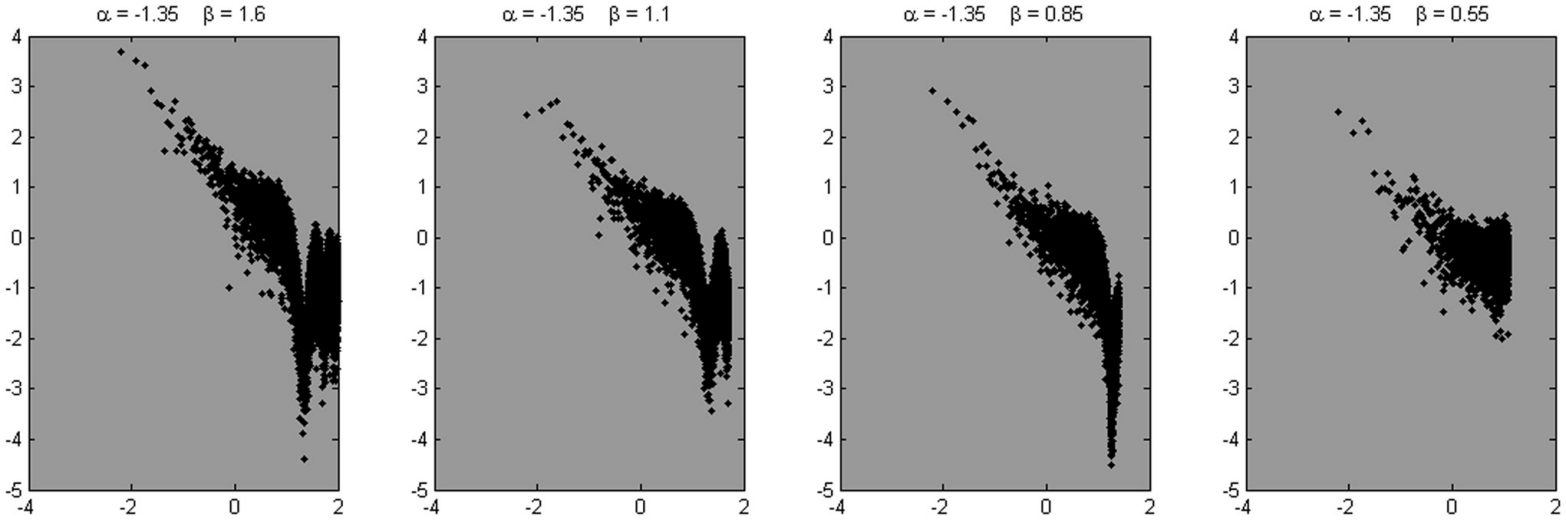
**Four example spectra are shown, derived from the signals simulated using the fBmW-model.** These signals were subjected to a moving average filter with a span of 14, 7, 4, and 2 data points, respectively, to mimic the reduced relative roughness at high sample rates. To these composites, another portion of white noise was added to mimic the flattening at the high-end of the power spectrum, as in Figures 5 and 6.

In search for a more realistic representation, we constructed 10 series with α = 1.35 with a series length of 2^16^, with white noise added to it (β = 1.6). The series were smoothed with a moving average filter with a span of 14 data points, to mimic the decrease in relative roughness at higher sample rates. This procedure added a steeper slope (i.e., lower amplitudes) at the high frequencies in addition to the initial flattening due to the added white noise. Then a portion of white noise was added again, to complete the staircase-shape of the empirical power spectra (i.e., white noise at the high-end of the power spectrum). Next, three times 10 series were produced, reducing in each case the series length and the number of overall estimated frequencies by a factor of two. Also the β parameter and the span of the moving average filter were reduced at each step. Examples of the resulting power spectra are shown in Figure 11.

**Figure 12:**
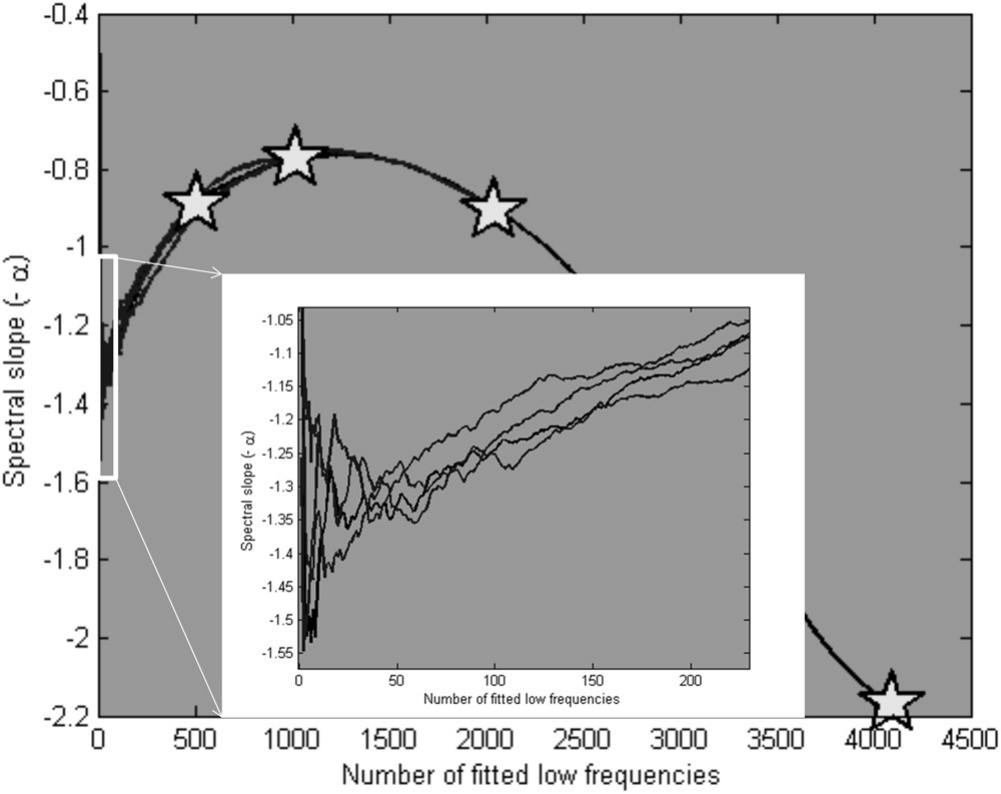
**Average scaling estimates of four times 10 simulated signals, with parameters presented in Figure 11, fitted over a varying number of low frequencies.** The pentagram markers indicate the scaling estimates for a fit over the 25% lowest frequencies. In the inset, it is shown that the estimates converge closely of the modeled α parameter (1.35) at around 50 low frequencies.

When fitted over a varying number of frequencies, the average estimate of 10 simulated series for each set of parameters converged on the “true” α of 1.35 at around 50 low frequencies. This can be seen in Figure 12 (see inset), which also shows the scaling estimates (as pentagram-shaped markers) when the lowest 25% of frequencies were used to fit the slope. Note that Figure 12 is restricted to 25% of low frequencies.

## General discussion

When spectral scaling exponents are estimated without anticipating artifacts introduced by sample rate, the exponent values themselves may fluctuate widely. The order of magnitude of these discrepancies is dramatic: scaling exponents may differ in magnitude by 1 or 2 depending on sample rate, while the order of magnitude of reliable differences in exponents between experimental groups and conditions are often in the range of 0.05–0.25 (e.g., Chen et al., 2001; Kello et al., 2007; Wijnants et al., 2009). These discrepancies may account for known inconsistencies in the psychological literature on 1/*f* noise, and perhaps, for the lack of a comprehensive framework of 1/*f* noise in continuous performance measures. Here we have introduced an empirical solution to this problem. The proposed strategy for spectral analysis is robust against changes in sample rate and renders more sensitive and valid α exponents compared with more conventional strategies of analysis.

The artifact introduced in the high-frequency range of a power spectrum by differences in sample rate is not due to the inherent difference in data series length (hence, Figure 7D) but is rather a natural consequence of the resulting differences in sample density. That is, denser sampling implies a decrease in relative roughness (i.e., because the highest frequencies in a measured signal have lower amplitude) compared with more sparsely sampled data. This artifact is important because it is implied that subtle methodological choices, often choices of convenience, may radically alter the outcome of spectral analysis when sampling artifacts are not adequately anticipated.

The proposed strategy for spectral analysis of continuous processes is to determine the spectral slope −α over a fixed number, rather than a fixed percentage of low-frequencies in a power spectrum. Fitting the slope over a set number of low frequencies implies a fit over a different high-frequency range for different sample rates, but over a stable low-frequency range. Fitting the slope over a fixed percentage of lowest frequencies, however, implies a fit over a stable high-frequency range, but over a different low-frequency range. Given that the artifact introduced by sample rate specifically concerns the high-frequency range of a power spectrum, it is obvious that the former strategy is to be preferred. That said, the aim of the present suggestion is not to exclude high-frequency range of a power spectrum *per se*, but rather to exclude comparisons that are unreliable in terms of frequency content (i.e., when a range of low frequencies quiescently varies in function of sample rate). While this may not solve the actual measurement problem (i.e., the outcomes change in function of measurement procedure and choices of data analysis), it does define the relation between observer and observable more clearly than before (i.e., outcomes should be independent of sample rate).

This suggestion follows the logic of Eke et al.'s (2002) recommendation to discard the highest frequencies and to focus on the lower frequencies, a recommendation that is consistent with all example studies cited in the section “1/*f* noise in human performance.” In the section “1/*f* noise and periodic sampling,” we acknowledged nonetheless that 1/*f* scaling relations often are observed within a finite range of scales only. The 1/*f* scaling relation may thus break down at specific frequency ranges, and usually at the highest frequencies. Interestingly, this basic fact about power spectra of psychological data series has led some scientists to inquire whether low- and high-frequency ranges in a power spectrum may represent the variability of different component mechanisms (Gilden, 2001; Delignières et al., 2008; Torre and Delignières, 2008). The scope of the present paper did not include an in depth discussion of that potential of spectral analysis. The present evaluation of spectral analysis reveals no reason to believe that such uses of spectral analysis are problematic in any way when dealing with trial series or simulated data series. Yet, the cautious implication is that estimating high-frequency slopes is a rather delicate enterprise when confronted with time series sampled at arbitrary sample rates.

The present investigation may shed new light on known discrepancies in the literature on 1/*f* noise in psychological data. For instance, an explicit demonstration of such a discrepancy is described by Delignières et al. (2005) in the context of a study of relative phase in bimanual coordination. These authors estimated the scaling properties of *discrete* relative phase, corresponding to a cycle-to-cycle measurement yielding a trial series. The mean values of the estimated scaling exponent α ranged from 0.72 to 0.78, while *continuous* relative phase (hence, the same performance when treated as a time series), results in scaling exponents with an average value of about 2.52 (Schmidt et al., 1991), far from the scaling range typically observed in trial series. This example confirms that different sampling regimes may effectively lead to appreciably different conclusions about the nature of the observed patterns of variability.

Also within a similar sampling regime (i.e., when an across-study comparison yields only time series, rather than comparing time series with trial series) different results may be obtained with different choices of sampling. An example is provided by studies of postural sway. “Postural sway typically exhibits fractal scaling with exponents characteristic of fractional Brownian motion (cf. Collins and De Luca, 1993), although prolonged, unconstrained standing has suggested a pink [*1/f*] noise structure (Duarte and Zatsiorsky, 2001)” (Bonnet et al., 2006, p. 806). These different results are methodologically interesting as well, if one notes that Collins and De Luca (1993) sampled their data at 100 Hz, while Duarte and Zatsiorsky (2001) sampled at 20 Hz. Here, we have shown that a comparison of these studies is only meaningful when the different sample rates of both experiments are taken into account, hence, when the scaling parameters are determined over an equivalent low-frequency range.

The ability to reliably and sensitively estimate valid scaling exponents, regardless of sample rate, and to compare these exponents (whether among different streams of 1/*f* noise, across experimental conditions or across studies) is undoubtedly a requisite to achieve a coherent and comprehensive framework of 1/*f* noise in continuous processes. The present contribution might motivate an extension of the coherent framework of 1/*f* noise that has emerged for trial series of repeated discrete responses (e.g., Diniz et al., 2010; Van Orden et al., 2011; Wijnants et al., 2012a,b) to continuous performance measures.

### Conflict of interest statement

The authors declare that the research was conducted in the absence of any commercial or financial relationships that could be construed as a potential conflict of interest.
